# Faecal Parasitology: Concentration Methodology Needs to be Better Standardised

**DOI:** 10.1371/journal.pntd.0004579

**Published:** 2016-04-13

**Authors:** Monika M. Manser, Agatha Christie Santos Saez, Peter L. Chiodini

**Affiliations:** UKNEQAS Parasitology, Department of Clinical Parasitology, Hospital for Tropical Diseases, London, United Kingdom; George Washington University, UNITED STATES

## Abstract

**Aim:**

To determine whether variation in the preservative, pore size of the sieve, solvent, centrifugal force and centrifugation time used in the Ridley-Allen Concentration method for examining faecal specimens for parasite stages had any effect on their recovery in faecal specimens.

**Methods:**

A questionnaire was sent to all participants in the UK NEQAS Faecal Parasitology Scheme. The recovery of parasite stages was compared using formalin diluted in water or formalin diluted in saline as the fixative, 3 different pore sizes of sieve, ether or ethyl acetate as a solvent, 7 different centrifugal forces and 6 different centrifugation times according to the methods described by participants completing the questionnaire.

**Results:**

The number of parasite stages recovered was higher when formalin diluted in water was used as fixative, a smaller pore size of sieve was used, ethyl acetate along with Triton X 100 was used as a solvent and a centrifugal force of 3,000 rpm for 3 minutes were employed.

**Conclusions:**

This study showed that differences in methodology at various stages of the concentration process affect the recovery of parasites from a faecal specimen and parasites present in small numbers could be missed if the recommended methodology is not followed.

## Author Summary

UKNEQAS Parasitology and participants themselves noted poor performance in examining faecal specimens that contained low numbers of parasites. Our survey of methods used showed that 96% of respondents used a concentration method based on the Modified Ridley-Allen technique, but there were differences regarding whether the formalin used was diluted in water or saline; the pore size of the sieve; the centrifugal speed; centrifugal time and the solvent used, depending on the commercial kit deployed. UKNEQAS conducted a study to investigate how differences in the various stages of the concentration method affected the recovery of parasites from faecal specimens. This study has shown that the preservative used, solvent used, differences in centrifugation time and speed, and pore size of the sieve all affect recovery. The modified Ridley Allen method was optimised when using ethyl acetate with triton X instead of ether and the centrifugation time was increased from one to three minutes. A concentration method is required for adequate detection of faecal parasites. Poor concentration technique leads to poor recovery of parasites. Variation in the procedure can reduce the recovery of parasite stages, particularly if present in small numbers.

## Introduction

The use of a concentration method to examine faeces for parasites increases the likelihood of finding ova, cysts and larvae, particularly in those specimens where they are present in insufficient numbers to be seen on direct microscopy. Concentration methods have been employed in clinical laboratories since 1948 when Ritchie [1] demonstrated their effectiveness. The method was improved by Ridley and Hawgood in 1956 [2] and simplified by Ridley and Allen in 1970 [3] using 10% formalin in water as a fixative, ether as a solvent to extract fat and debris, followed by filtration through a sieve with a pore size of 425 microns and centrifugation at 3000rpm for 1 minute to leave the ova, cysts and larvae in the sediment at the bottom of the centrifugation tube. The method uses several pieces of apparatus which are washed after the concentration of each sample and also has health and safety implications since formalin is an irritant and ether is flammable. The hazardous aspects and labour-intensive nature of the method encouraged commercial companies to promote enclosed, disposable faecal concentration systems which claim to have comparable sensitivity to the Ridley Allen Method in recovering parasites. These kits reduce the hazards of formalin by being enclosed and by using ethyl acetate rather than ether, since it is less flammable and more stable. Young *et al* [4] showed ethyl acetate to be an acceptable alternative to ether in the recovery of parasites.

In 2011, UKNEQAS Parasitology sent a questionnaire to all participants of the Faecal Parasitology Scheme to ask for their routine method for concentrating faecal samples for the detection of parasites. This was in response to concerns expressed by UKNEQAS Parasitology who noted poor performance among participants examining faecal specimens that contained low numbers of parasites. Those concerns were also raised by participants themselves who reported recovering lower numbers of parasites than those seen by UKNEQAS Parasitology in the pre-distribution examination of the EQA specimens. Furthermore, where parasites were present in low numbers they were failing to see them at all, thus adversely affecting their score in the NEQAS Faecal Parasitology Scheme. Following an analysis of the questionnaire it was apparent that although 96% of respondents used a concentration method based on the Modified Ridley Allen [3] technique, there were differences regarding whether the formalin used was diluted in water or saline; pore size of the sieve (0.35mm– 1.5mm or no sieve used); the centrifugal speed (500–3,500 rpm); centrifugal time (1–10 minutes); and the solvent used (ether, ethyl acetate or no solvent) depending on the commercial kit deployed. These findings prompted UKNEQAS to conduct a study to investigate how differences in the various stages of the concentration method affected the recovery of parasites from faecal specimens.

## Materials and Methods

For this study we used the following chemicals and kits: Ether (VWR International), Ethyl acetate (VWR International), Formalin (VWR International), Triton X 100 (Sigma Aldrich) and Midi Parasep faecal parasite concentrator (Apacor).

### Questionnaire

A questionnaire to ascertain the concentration methods routinely used in clinical laboratories to recover parasites from faecal specimens was sent to all 580 participants in the UKNEQAS Faecal Parasitology Scheme. Two hundred participants returned a completed questionnaire. The questionnaire sought information on the kit, fixative, pore size of the sieve, solvent, plus the centrifugation speed and time used to deposit the parasites. After analysis of the participants’ responses, UKNEQAS Parasitology conducted a study to examine those aspects of the concentration method that deviated from the modified Ridley Allen method. The stages examined were:

### Comparison between formalin diluted in water and formalin diluted in saline (fixative)

Twenty specimens preserved in 10% formalin in water were concentrated using 10% formalin in water or 10% formalin in saline as a fixative.

### Comparison of 3 different pore sizes

Eight specimens preserved in 10% formalin and water were examined using 3 different pore sizes of sieve; 425μm, 800μm and 1,500μm.

### Comparison of ether and ethyl acetate

Fifty four specimens preserved for between 6 months and 2 years in 10% formalin and water and 24 fresh unfixed specimens containing ova, cysts and larvae were concentrated and examined to compare the recovery of parasites using ether or ethyl acetate as an extractor of fat and debris. Since the polarity and miscibility indices [5] of ether result in its being a better fat extractor than ethyl acetate, the surfactant Triton X 100 is added to the faecal/formalin solution to compensate if ethyl acetate is used. Triton X 100 helps ethyl acetate to break up faecal matter and results in a less dense deposit, thus facilitating the identification of parasite stages. A concentration of 0.1% Triton X 100 i.e. 1 mL added to 1 litre of formalin/water was noted to be the most effective concentration since excess Triton X 100 results in a soapy deposit which makes it difficult to examine. The effect of not using a solvent was not examined in this study as it has already been shown that the recovery of parasite stages is significantly lower and more debris is present if a solvent is not used [3].

### Comparison of centrifugal speed and centrifugal time

Eight samples preserved in 10% formalin and water were examined to assess the effect of parasite recovery using different centrifugation times, 1 minute, 2 minutes, 3 minutes, 4 minutes, 5 minutes and 10 minutes and different centrifugation speeds (forces), 500rpm (34G), 1000rpm (134G), 1,500rpm (300G), 2000rpm (537G), 2,500rpm (840G), 3,000rpm (1,200G) and 3,500rpm (1643G). The centrifugal times and speeds were selected in accordance with those used by participants as reported on the returned questionnaires. All centrifugations were carried out on the same centrifuge.

### Concentration method

The Midi Parasep faecal parasite concentrator, an enclosed system that employs the principle of the Ridley-Allen formol-ether sedimentation technique was used to concentrate samples for the comparison of ether and ethyl acetate [6]. A pea-sized amount (equivalent to approximately 1 gram) of faeces was mixed with 6 millilitres (mL) of 10% formalin in water in the mixing chamber. Two mL of ether or ethyl acetate was added (formalin/Triton-X 100 mixture was added to the mixture if ethyl acetate was used as it helps to emulsify the faecal matter). Parasep was assembled and sealed by screwing the filter thimble and sedimentation cone onto the mixing chamber. The mixture was vortexed for 15 seconds and the system inverted to allow the mixture to be filtered through the filter thimble (pore size of 425μm) and centrifuged at 1200g or 3000 rpm for 1–3 minutes according to the manufacturer’s instructions at the time. The mixing chamber and the filter thimble were unscrewed together and discarded. Like the conventional Ridley-Allen sedimentation method, there is an upper ethyl acetate layer, fatty plug, formalin supernatant and deposit. The fatty plug was loosened and the supernatant was safely discarded according to the Control of Substances Hazardous to Health regulations 2002 [7]. The concentration procedure apart from the centrifugation stage was undertaken in a Class 1 safety cabinet.

The Parasep faecal parasite concentrator with ethyl acetate as a solvent and Triton X 100 as a surfactant was used to compare the different centrifugal speeds and times. The recommended speed of 3000rpm and time of 1–3 minutes were altered according to the variations in speed and time quoted by participants.

The Parasep faecal parasite concentrator with ethyl acetate as a solvent and Triton X 100 as a surfactant according to the manufacturer’s instructions was used to compare 10% formalin diluted in water and 10% formalin diluted in saline as a preservative.

The conventional Ridley-Allen method with ethyl acetate and Triton X as a solvent was used to compare the sieves with different pore sizes.

Prior to the microscopic examination, all faecal deposits were re-suspended in 75μL of saline (three drops) and thoroughly mixed. In each case, 50μL of the diluted deposit was dispensed onto a microscope slide and a 22mm by 22mm coverslip applied.

The whole of the coverslip was examined and the number of ova, cysts and larvae recorded. All specimens were processed in duplicate and the mean calculated.

### Statistical analyses

The Wilcoxon Matched-Pairs Signed-Ranks Test which uses the sizes of the differences was the statistical test used to compare the recovery of parasites in formalin in water versus formalin in saline, ether versus ethyl acetate and the different pore sizes of the sieves.

## Results

### Analyses of the questionnaire

**Fig 1:**
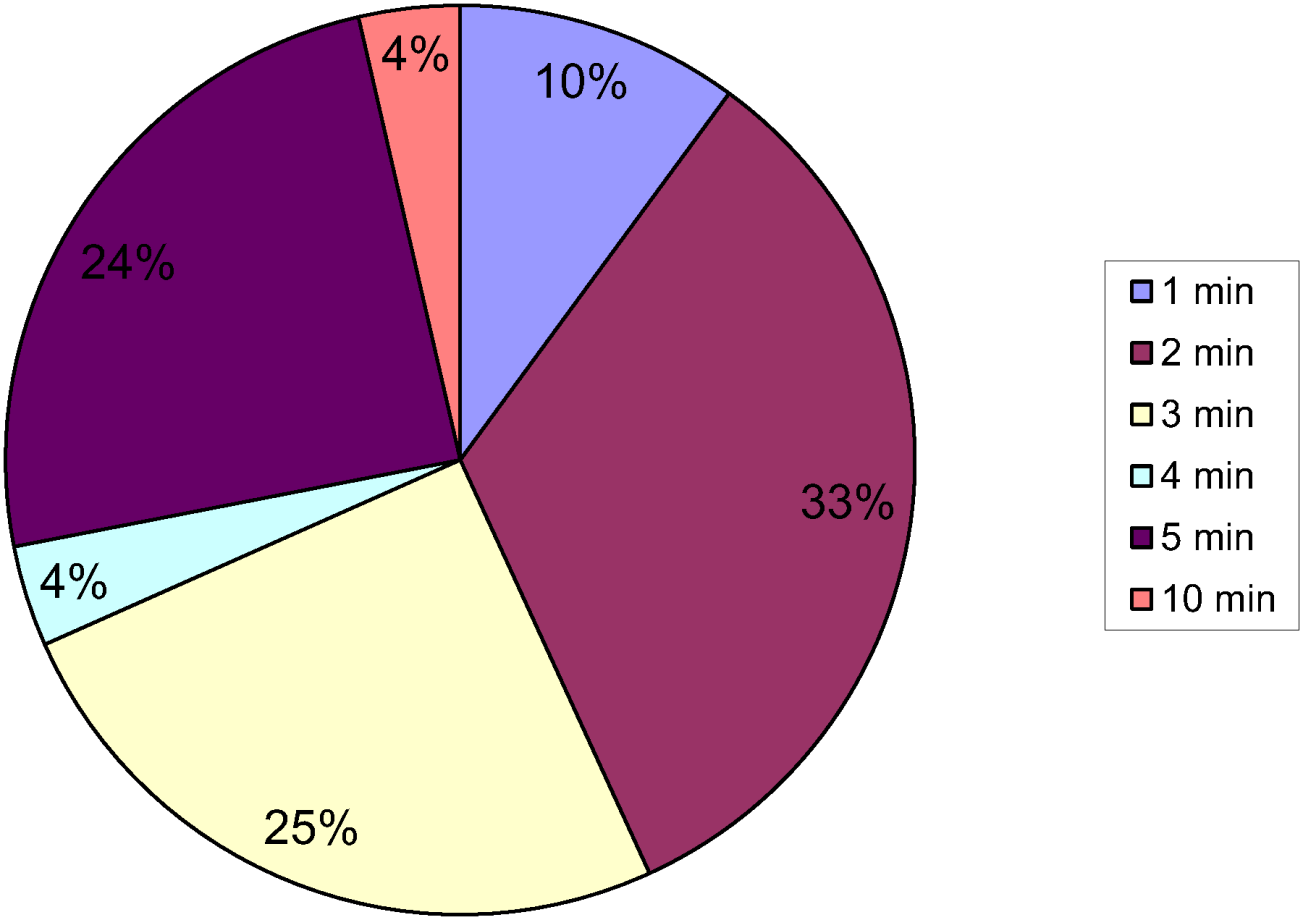
Centrifugation time.

**Fig 2:**
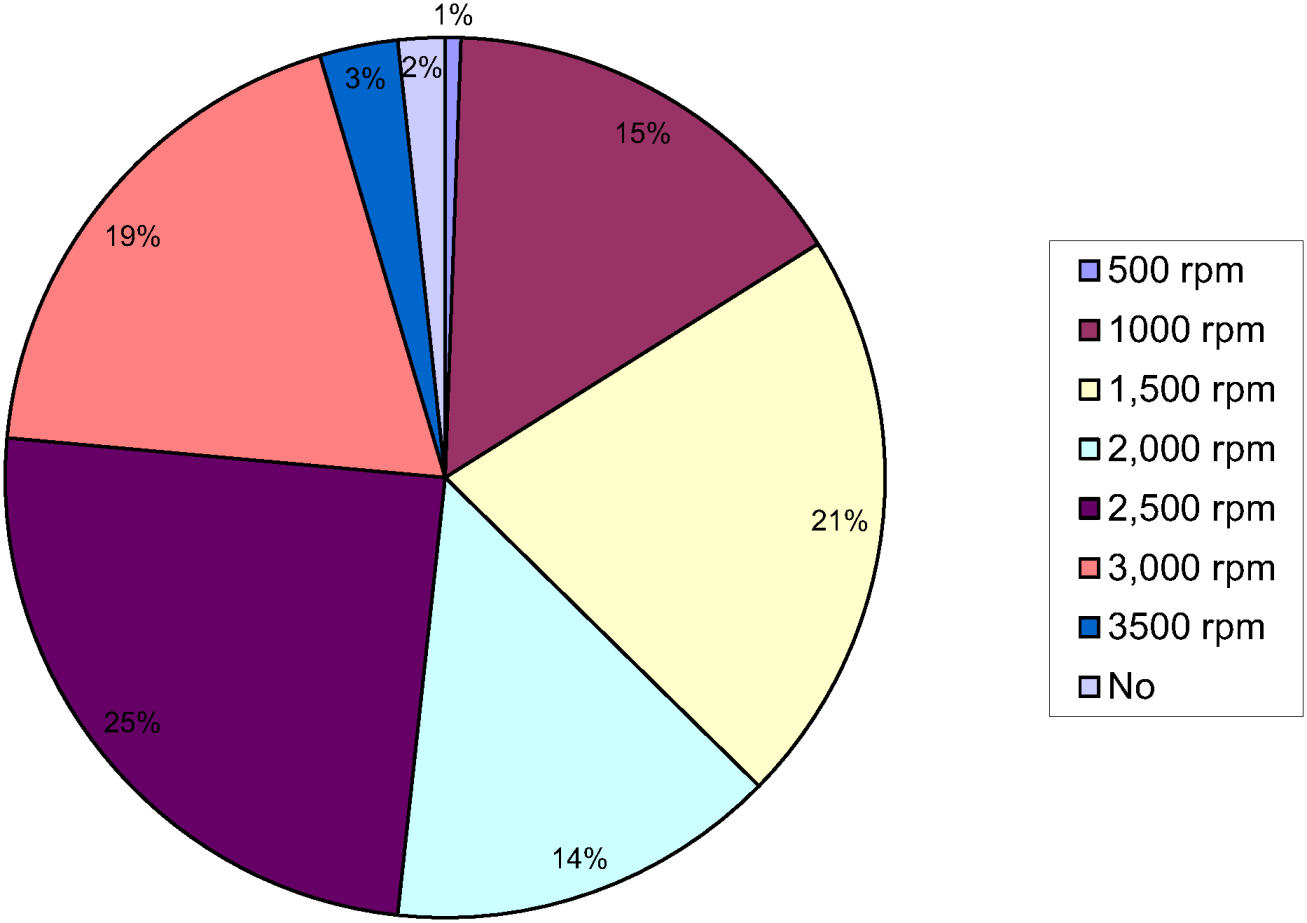
Centrifugation speed.

The commercial kits used by participants, the manufacturer’s recommended procedure, the procedure used by participants, and the number of participants who followed the manufacturer’s procedure are shown in Table 1. The centrifugal speeds and times used by participants are shown in Figs 1 and 2 respectively.

**Table 1:** Analyses of participants’ concentration procedures.

		Analyses of participants’ procedures										
		Preservative *						Solvent **				
Manufacturer	Manufacturer’s instructions	FW	FS	SAF	PVA	MIF	Ecofix	Et	EtA	EtA +TrX	None	No of participants who followed manufacturer’s procedure for centrifugal speed and time
Modified Ridley-Allen N = 57	10% formalin/water, Ether as solvent, Centrifuge at 3000 rpm for 1 min.	21	10	16	2	1		19	25	2		**6** (for the remainder, centrifugal speed ranged from 500–3,500 rpm and time from 1–10 mins
Midi Parasep N = 59	10% formalin/water, Ethyl acetate/Triton X, Centrifuge at 3000 rpm for 1–3 minutes	15	27	10				2	23	24		**22** (for the remainder, centrifugal speed ranged from 500–3,000 rpm and time from 1–5 mins)
Midi Parasep SF N = 24	10% formalin/water, SAF,PVA,MIF; No solvent; Centrifuge for 1,400 rpm for 3 minutes	8	7	9							24	**14** (for the remainder, centrifugal speed ranged from 500–3000 rpm and time from 1–3 mins)
Mini Parasep SF N = 3	10% formalin/water,SAF,PVA,MIF; No solvent; Centrifuge for 200g for 3 minutes	3									3	Manufacturer’s instructions not followed (Centrifugal speed ranged from 1,200–2,500 rpm and time from 1–2 mins)
FPC Evergreen N = 17	10% formalin/water, Ethyl acetate, Centrifuge at 500g for 10 mins	5	4	8				5	3	2	1	**4** (for the remainder, centrifugal speed ranged from 1,200–2,500 rpm and time from 1–10 mins)
Parapak N = 15	Formalin/water, PVA, SAF or Ecofix, Ethyl acetate, 1,800–2,200 rpm for 10 mins	4	1	5		1	1	1	9	1	2	**5** (for the remainder, centrifugal speed ranged from 1,200–3,000 rpm and time from 1–5 mins)
Diamondial N = 8	Formalin/water, PVA, SAF or Ecofix, Ethyl acetate, 1,000g for 10 mins			2					4			**4** (for the remainder, centrifugal speed ranged from 3,000–3,500 rpm and time from 1–5 mins)
Easy Copros N = 2	Formalin/water, ethyl acetate, 1,500rpm for 5 mins.		1						1			1 participant did not state speed and time the other centrifuged at 1,500 rpm for 10 mins.
Storax N = 1	Manufacturer’s instructions not found			1				1				Participant centrifuged at 1,800 rpm for 10 mins.
Para-Sed MCC N = 1	Formalin/water, SAF,ethyl acetate/triton X, 500g for 10 mins			1				1				Participant followed manufacturer’s instructions
Biosepar	Medium FixSepar ECO, 1000g for 5 minute			1					1			Participant centrifuged at 1,500 rpm for 2 mins
In-house N = 8		2	2	3				1	3			Centrifugal speeds ranged from 500–3,000 rpm and time 1–10 mins.
No concentration method N = 7												Direct Examination only

*The preservatives used are as follows: FW, 10% formalin in water; FS, 10% formalin in saline; SAF, Sodium acetic acid acetate formalin; MIF merthiolate Iodine formalin; PVA Poly vinyl alcohol.

** The solvents used are as follows: Et, ether; EtA, ethyl acetate; EtA TrX, ethyl acetate + TritonX

Numbers of participants using solvent and preservative do not equate to the total number of participants using the particular test because many participants did not state what preservative or solvent they used.

### Comparison between formalin diluted in water and formalin diluted in saline

The number of ova and cysts for each parasite species in individual specimens when 10% formalin in water or 10% formalin in 0.9% saline was used as a fixative are shown in Table 2. The Wilcoxon Matched-Pairs Signed-Ranks Test showed that formalin in water was significantly more effective than formalin in saline in the recovery of parasites (p < = 0.005).

**Table 2:** Comparison of the recovery of parasites using formalin in water and formalin in saline.

Specimen content (n = number of specimens containing the parasite)	Formalin in water	Formalin in saline
***Ascaris lumbricoides (*n = 2*)***	175, 211	75, 99
***Trichuris trichiura (*n = 2*)***	23, 525	10, 343
***Hookworm species (*n = 3*)***	6, 8, 7	4, 2, 6
***Taenia species***	110	24
***Capillaria philippinensis***	28	16
***Diphyllobothrium latum***	149	81
***Fasciola species***	33	30
***Schistosoma mansoni***	13	12
**L3 larvae of *Strongyloides stercoralis***	36	30
***Entamoeba histolytica/dispar (*n = 2)**	80, 40	47, 5
***Entamoeba coli***	136	62
***Endolimax nana***	130	71
***Blastocystis hominis***	101	60
***Chilomastix mesnili***	113	84
***Giardia intestinalis***	389	136

The Wilcoxon Matched-Pairs Signed-Ranks Test: p < = 0.005

### Comparison of 3 different pore sizes

The number of ova recovered using 3 different pore sizes of sieve during the filtration stage are shown in Table 3. There was a significant difference in the recovery of parasites between the 3 different sieves. The pore size of 425 μm, as recommended in the Ridley-Allen Method resulted in the best recovery of ova (Table 3). The amount of debris seen in the deposit increased with pore sizes above the recommended, making ova more difficult to see.

**Table 3:** Comparison of the recovery of parasites using sieves of 3 different pore sizes.

	No of ova per coverslip		
Specimen Content	Sieve 1	Sieve 2	Sieve 3
	Parasep (425μm)	(800 μm x800 μm)	(2,000 μm x 1,000 μm)
***Ascaris lumbricoides***	453	355	36
***Hookworm sp***	27	21	2
***Fasciola sp*.**	20	15	12
***Schistosoma mansoni***	10	5	2
***Trichuris trichiura***	51	43	6
***Hymenolepis nana***	5	1	0
	**Sieve 1 vs Sieve 2**	**Sieve 1 vs Sieve 3**	**Sieve 2 vs Sieve 3**
**The Wilcoxon Matched-Pairs Signed-Ranks Test**	p< = 0.005	p< = 0.005	p< = 0.005

### Comparison of ether and ethyl acetate

The number of ova and cysts for each parasite species in each specimen (some parasites were present in multiple specimens) are shown in Table 4 for the formalin-preserved specimens and Table 5 for the fresh, unpreserved samples.

**Table 4:** Ether vs ethyl acetate: Comparison of the recovery of parasites preserved in formalin.

	Average number of parasites per coverslip	
Specimen content (n = number of specimens containing the parasite)	Ether	Ethyl acetate
***Ascaris lumbricoides* (n = 7)**	180, 145, 0, 38, 186, 152, 0	206, 115, 2, 273, 148, 192, 2
**Hookworm species (n = 8)**	202, 121,110, 50, 6, 50, 5, 5	220, 132, 106, 70, 7, 60, 12, 4,
***Trichuris trichiura* (n = 6)**	16, 8, 9, 16, 10, 27	19, 12, 1, 36, 16, 33
***Toxocara canis* (n = 1)**	12	198
***Taenia species* (n = 1)**	28	80
***Diphyllobothrium latum*(n = 1)**	45	55
***Fasciola sp*. (n = 1)**	30	46
***Capillaria philippinensis* (n = 1)**	150	131
**Rhabditiform larvae of *Strongyloides stercoralis*(n = 3)**	40, 32, 9	27, 17, 10
***Schistosoma mansoni* (n = 2)**	12, 10	10, 17
***Hymenolepis nana* (n = 3)**	28, 2, 0	125, 2, 5
***Giardia intestinalis* (n = 6)**	3, 10, 2, 9, 0, 1	37, 58, 68, 9, 104, 14
***Entamoeba histolytica/dispar (n = 2)***	3, 4	4, 4
***Endolimax nana* (n = 4)**	1540, 1038, 0, 20	1620, 1976, 40, 596
***Entamoeba coli (n = 4)***	28, 14, 20, 35	20, 12, 15, 25
***Cyclospora cayetanensis (n = 3)***	3, 12, 1	7, 9, 2
***Isospora belli* (n = 1)**	28	22

The Wilcoxon Matched-Pairs Signed-Ranks Test: p < = 0.001036

**Table 5:** Ether vs Ethyl acetate: Comparison of the recovery of parasites in fresh unpreserved specimens.

Specimen content (n = number of specimens containing the parasite)	Average number of parasites per coverslip	
	Ether	Ethyl acetate
***Ascaris lumbricoides* (n = 1)**	52	80
***Trichuris trichiura* (n = 1)**	41	26
**Rhabditiform larvae of *Strongyloides stercoralis* (n = 1)**	18	16
***Taenia species* (n = 1)**	6	40
***Hymenolepis nana* (n = 3)**	2, 10, 12	40, 50, 65
***Giardia intestinalis* (n = 5)**	2, 17, 10, 0, 2	1, 16, 18, 6, 36
***Endolimax nana* (n = 2)**	540, 644	1530, 1940
***Entamoeba coli* (n = 4)**	36, 16, 27, 460	18, 4, 21, 540
***Iodamoeba butschlii* (n = 2)**	35, 700	247, 740
***Entamoeba histolytica/dispar* (n = 4)**	9, 252, 32, 240	11, 340, 28, 330

Use of ethyl acetate and Triton X recovered more parasites in both the preserved (p < = 0.001036) and unpreserved specimens (p < = 0.003732) than using ether. Although ether recovered more parasites than ethyl acetate and Triton X 100 from 11/54 preserved samples and 8/24 fresh unpreserved samples, ether failed to recover parasites from 5/54 preserved samples and 1/24 fresh unpreserved samples, whereas use of ethyl acetate and Triton X 100 recovered parasites from all of them. When using ether, ova and cysts became trapped in the fatty layer and were consequently disposed of with the supernatant, though the deposit contained less debris than when ethyl acetate was used.

### Comparison of centrifugal speed and centrifugal time

**Fig 3:**
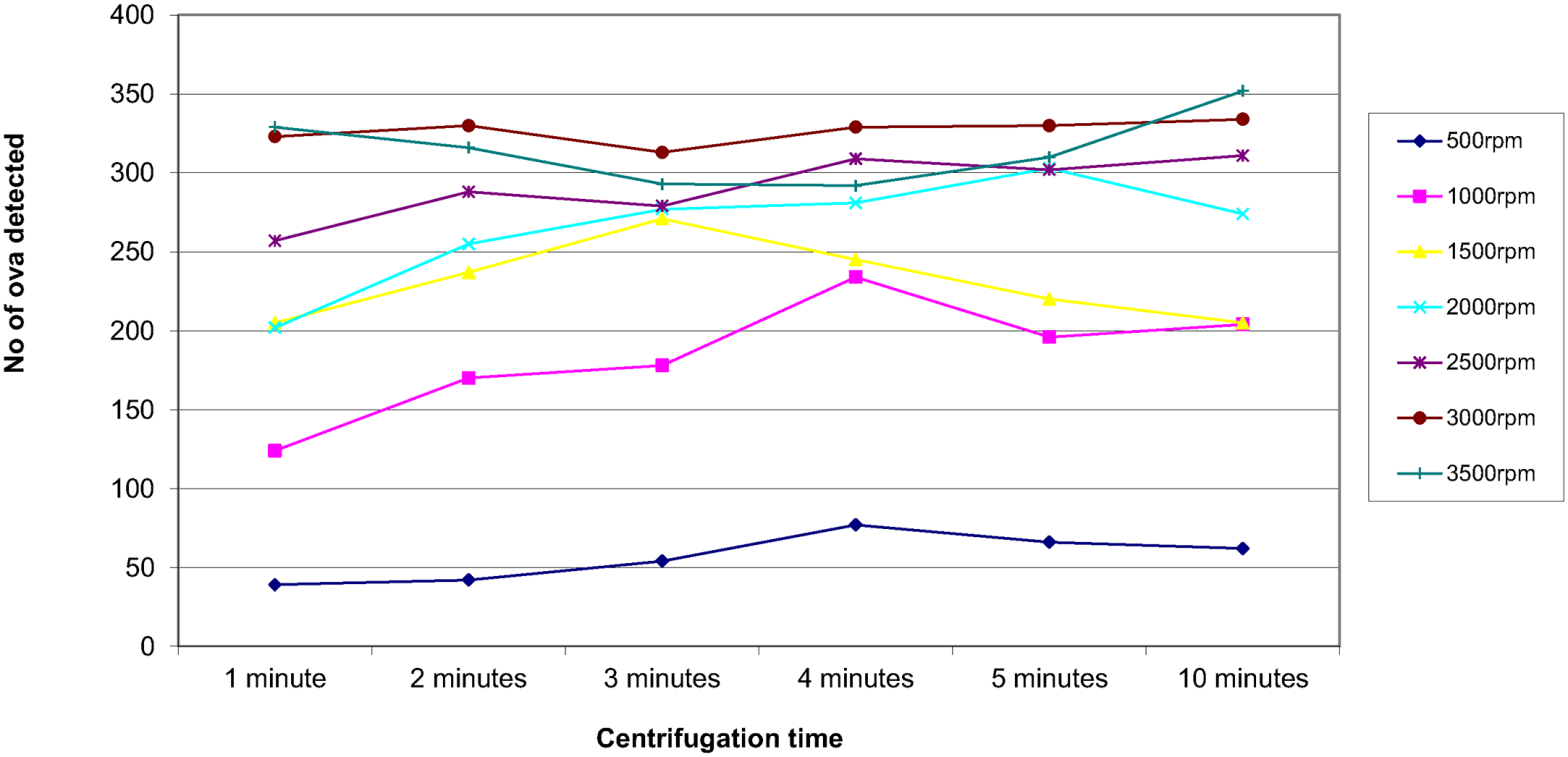
Recovery of *Ascaris lumbricoides* based on centrifugal speed and time.

**Fig 4:**
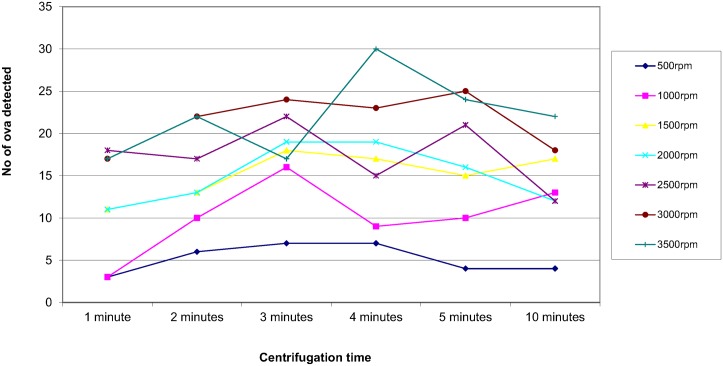
Recovery of *Trichuris trichiura* based on centrifugal speed and time.

**Fig 5:**
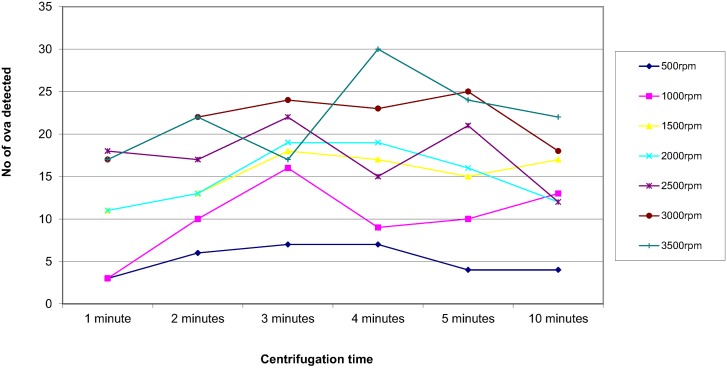
Recovery of Hookworm species based on centrifugal speed and time.

**Fig 6:**
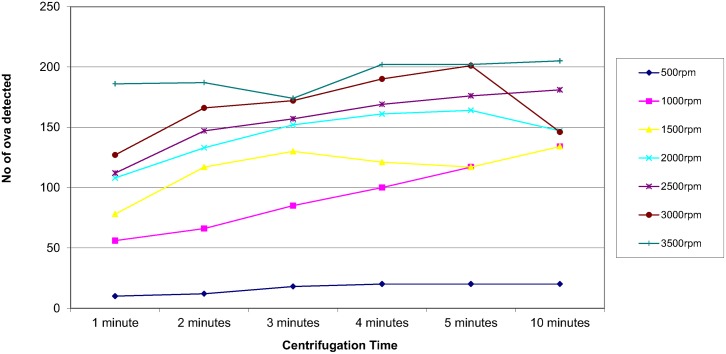
Recovery of *Taenia* species based on centrifugal speed and time.

**Fig 7:**
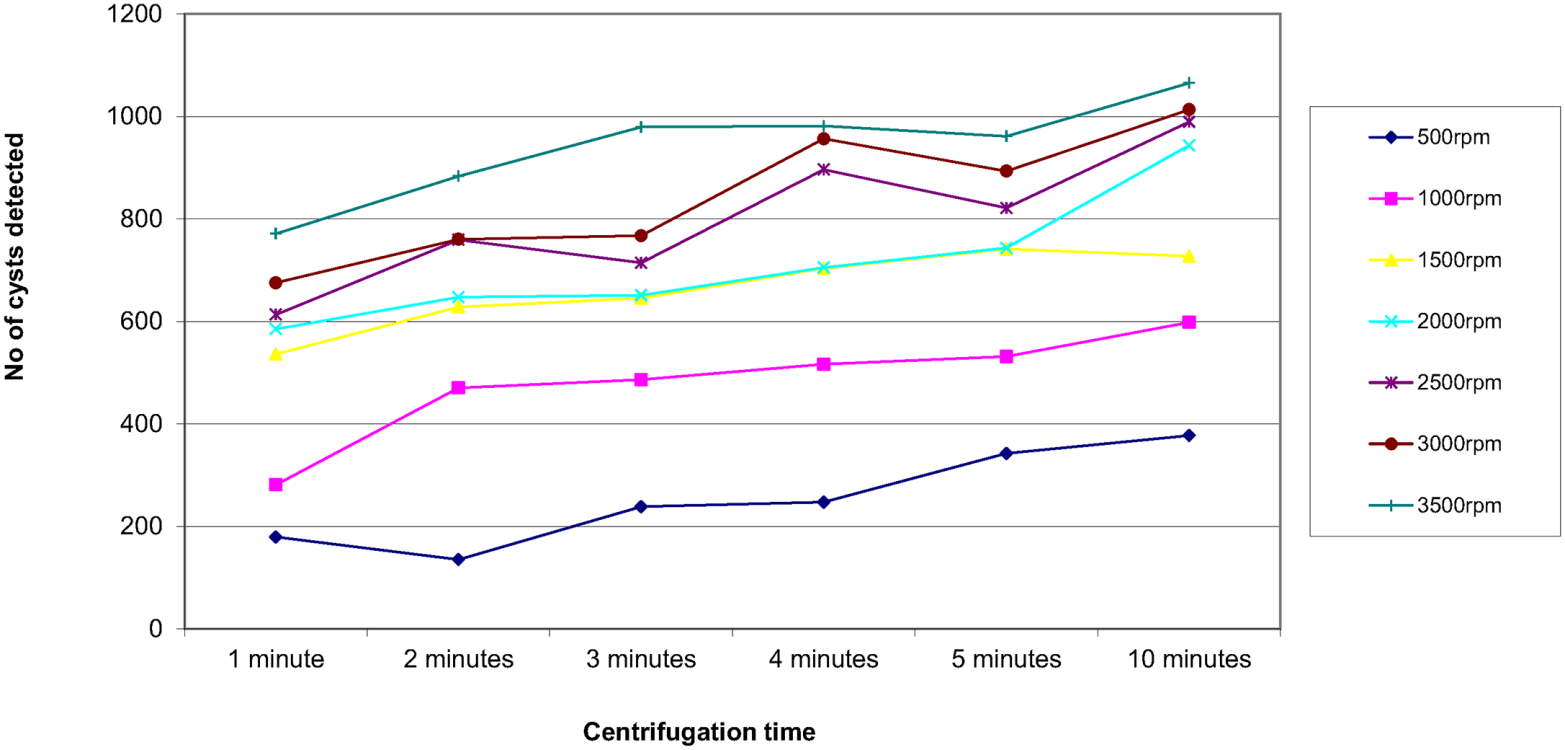
Recovery of *Entamoeba coli* based on centrifugal speed and time.

**Fig 8:**
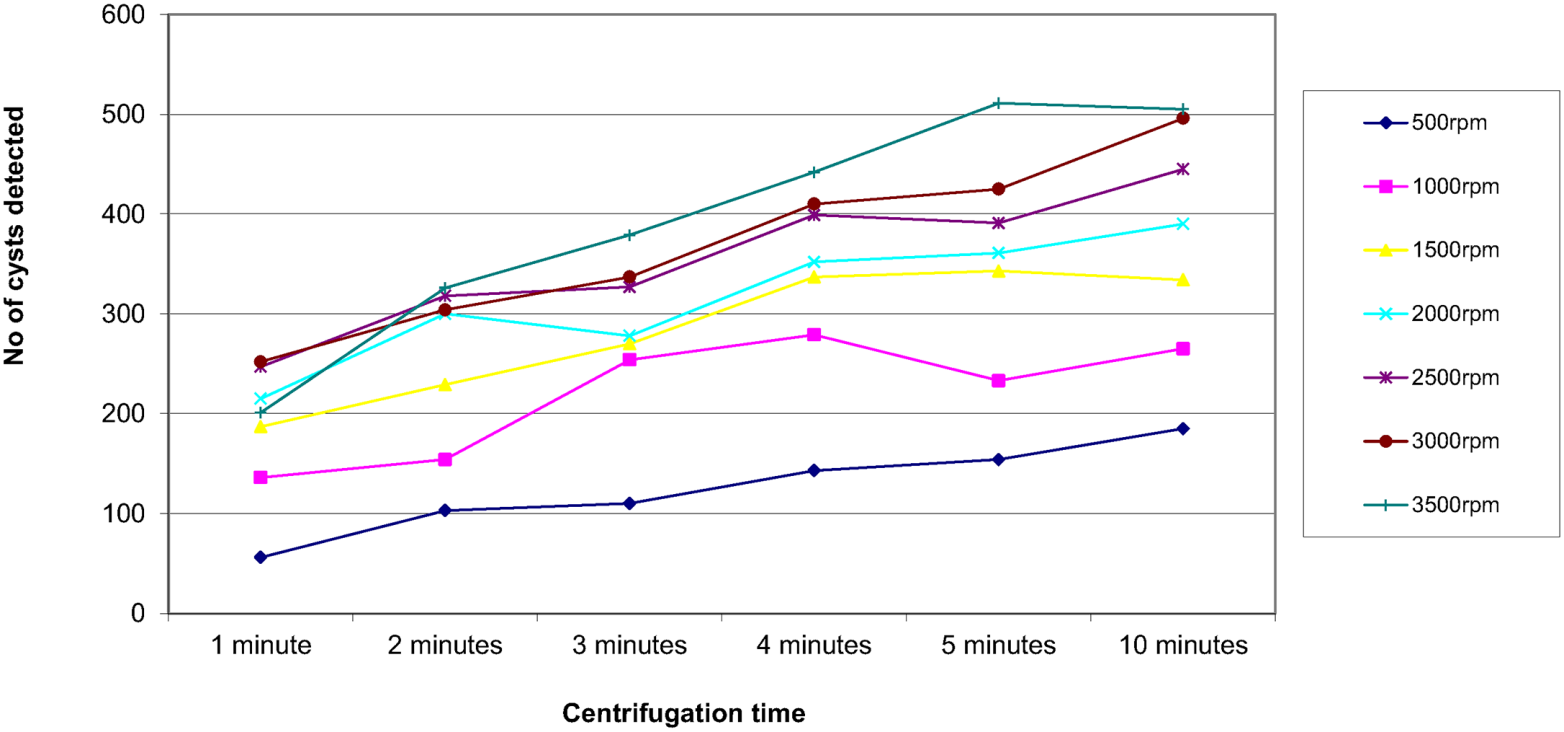
Recovery of *Entamoeba histolytica/dispar* based on centrifugal speed and time.

**Fig 9:**
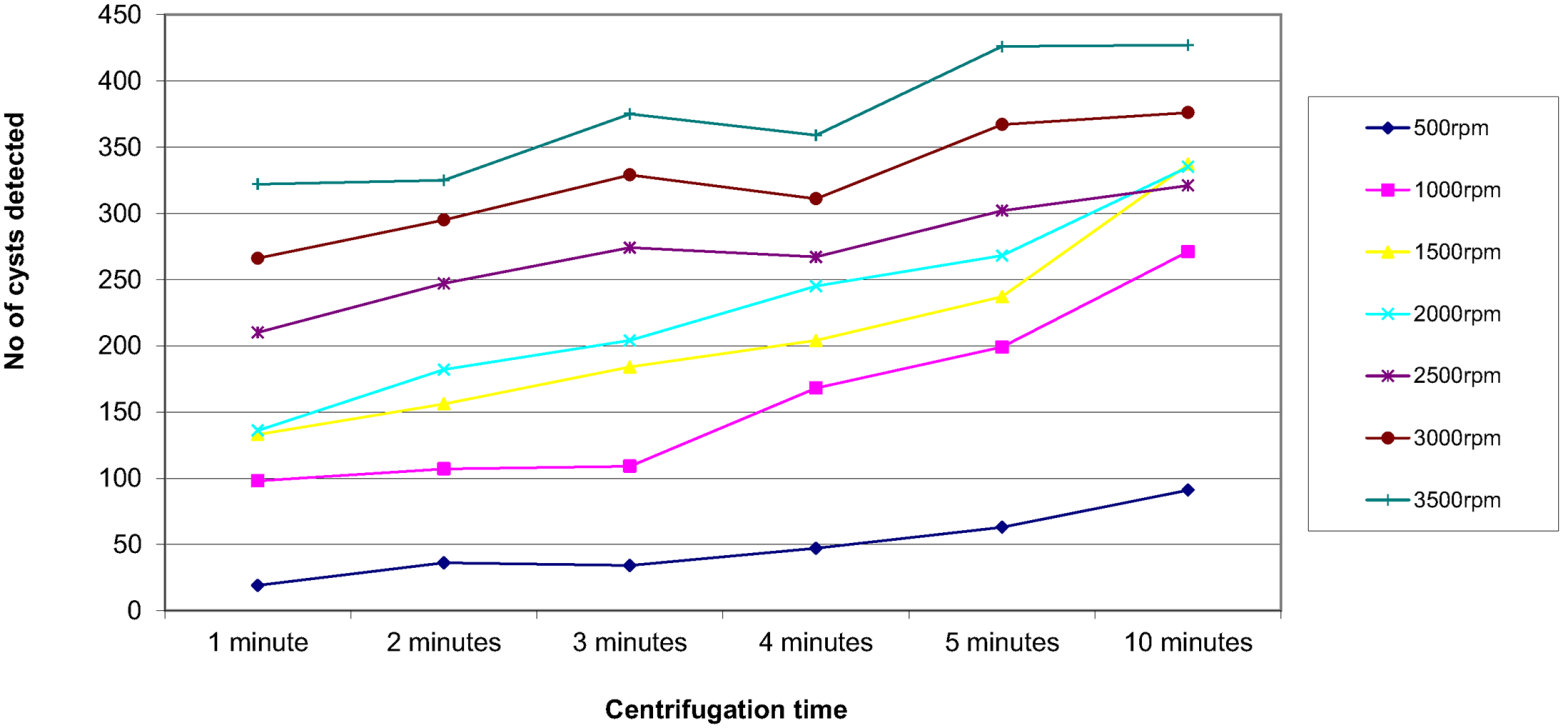
Recovery of *Entamoeba hartmanni* based on centrifugal speed and time.

**Fig 10:**
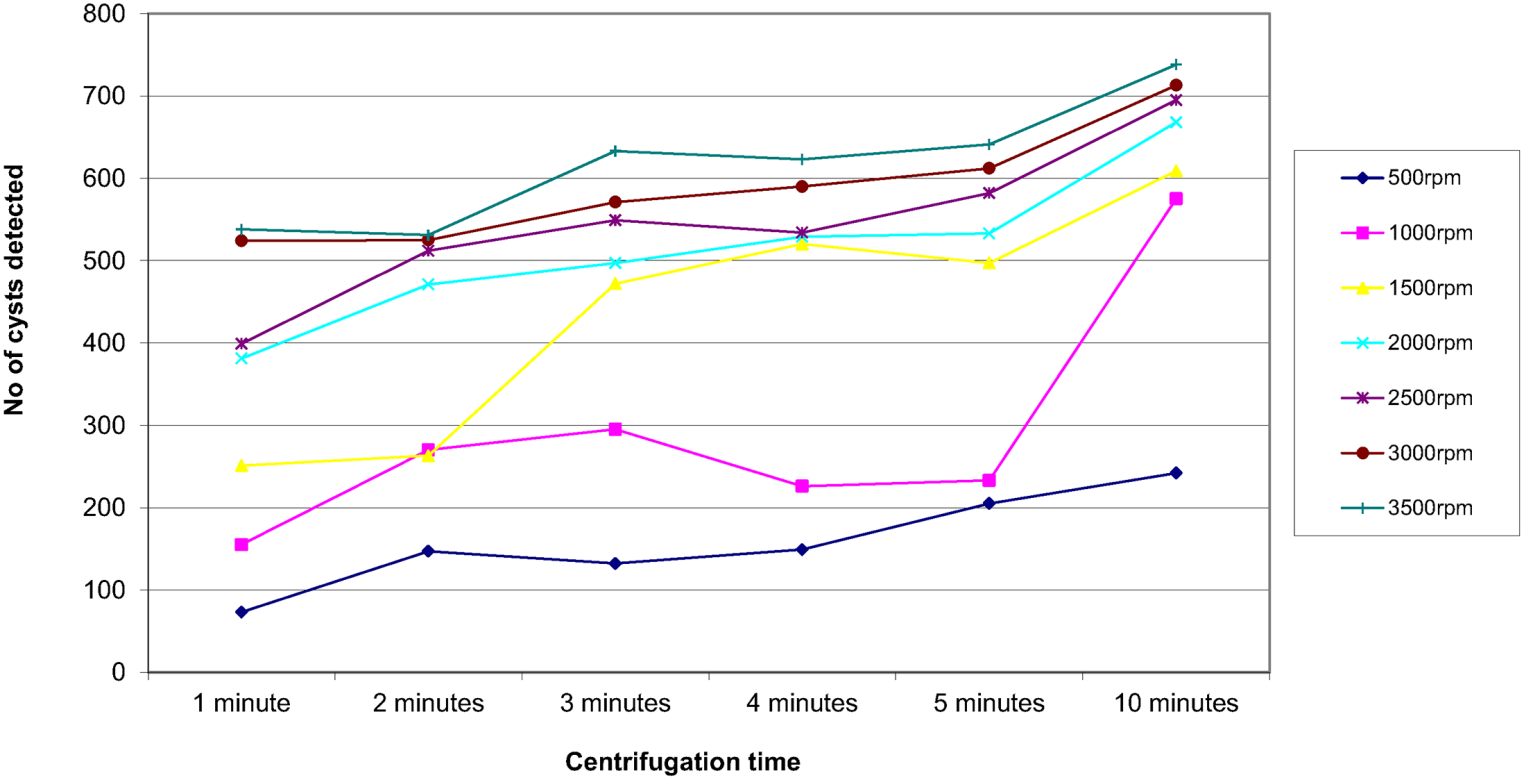
Recovery of *Endolimax nana* based on centrifugal speed and time.

In order to assess the effect of centrifugal speed and time on the recovery of parasites, 8 organisms were selected; *Ascaris lumbricoides*, *Trichuris trichiura*, Hookworm species, *Taenia* species, *Entamoeba coli*, *Entamoeba histolytica/dispar*, *Entamoeba hartmanni* and *Endolimax nana*. They were concentrated using the different speed and time combinations reported in the questionnaire. The results are shown in Figs 3–10. The number of parasite stages detected increased with an increase in centrifugal speed and time. However, although the recovery of parasites was greatest if the samples were centrifuged for 3,500rpm for 10 minutes, the deposit was more difficult to examine due to excess debris masking the parasites present.

## Discussion

The use of a concentration method is essential for the examination of faeces for parasitic diseases as it increases the likelihood of finding ova, cysts and larvae, particularly in those specimens where they are present in numbers too low to be seen by direct microscopy. The modified Ridley-Allen formol-ether concentration technique [3] is the method of choice for routine use by most clinical laboratories and is the method on which most commercial kits are based. This procedure entails filtration of a faecal suspension in 10% formalin in water, solvent extraction of debris and fat, followed by centrifugation.

This study examined the concentration methods reported by participants in the UK NEQAS Faecal Parasitology Scheme. We assessed the recovery of parasites in the 4 stages of the concentration procedure that showed the most variation in methodology reported by participants: the solvent used, the sieve pore size, the fixative used, and the centrifugation speed and time, to establish if recovery of parasites was affected by the different methods.

Although most of the parasites were seen in the deposits obtained using all the different methods used, significantly fewer ova and cysts were recovered using 10% formalin in saline rather than in water, ether rather than ethyl acetate, lower centrifugal speeds and times and a larger pore size of filter.

Many years ago (1970), Ridley and Allen showed that formalin in water was more effective in recovering parasites than formalin in saline, which had been used in the Ridley and Hawgood technique of 1956 [2]. This is due to formalin in saline being denser than formalin in water so the lighter parasites tend to stay in solution or become trapped in the fatty layers and risk being discarded along with the fatty plug if saline is used.

When comparing the 3 different pore sizes of sieve used to filter the faecal specimen, the larger the pore size, the more debris was retained, making the deposit much thicker and more difficult to examine since the ova or cysts were being masked by excess debris. In order to facilitate microscopic examination, more saline had to be added to the deposit to dilute the specimen such that the recovery of parasites per coverslip was significantly less (p< = 0.005). Both Ridley and Hawgood [2] and Allen and Ridley [3] recommended a sieve with a pore size of 425microns. Sieving of the faeces and formalin mixture prior to centrifugation is important in eliminating large pieces of faecal matter from the suspension.

When comparing ether and ethyl acetate, both retain the morphology of most ova, cysts and parasites. Although ethyl acetate is less flammable, less combustible and more stable than ether, it results in a thicker deposit. However the addition of Triton X 100 to the faecal formalin solution along with ethyl acetate helps to break up faecal matter and results in a less dense deposit, facilitating the identification of parasite stages. Ethyl acetate and Triton X100 recovered significantly more ova and cysts from both preserved (p < = 0.001036) and fresh samples (p < = 0.003732) than ether, an observation also made by Young et al in1979 [4]. In addition, using ether as a solvent on 5 preserved samples, ova of *Ascaris lumbricoides* were missed in 2 specimens, *Hymenolepis nana*, *Endolimax nana* and *Giardia intestinalis* were missed in one each of the specimens and in 1 fresh sample, cysts of *Giardia intestinalis* were missed when using ether. In those specimens where ether recovered more parasites than ethyl acetate i.e. 11/54 preserved samples and 8/24 fresh samples, there were sufficient numbers of parasites seen in the ethyl acetate deposit that they would not have been missed by the microscopist. Although the use of ether as a solvent resulted in a deposit free of fat and debris, ova and cysts became trapped in the fatty plug and discarded along with the supernatant. This is due to the different chemical characteristics of ether and ethyl acetate, ethyl acetate being more polar [5] than ether, making ether better for emulsifying fats but on the other hand attracting some ova and cysts whose cell membranes have a high fat content, resulting in their becoming trapped in the fatty layer. Ethyl acetate is a less effective emulsifier of fats and is thus better for depositing ova and cysts, at the cost of more debris being present in the deposit.

Regarding centrifugal speed and time, the number of parasite stages recovered increased with an increasing speed and time. In some concentration kits, the manufacturer’s instructions advise centrifuging on a low speed for a longer time to prevent destroying the parasite stages, Figs 3–10 demonstrate that an adequate centrifugal force must be used to sediment the ova and cysts. Centrifuging at a lower speed for a longer time resulted in significantly lower recovery of ova and cysts than centrifuging at the recommended 3000 rpm for 1 minute. This supports the observation made by Ridley and Allen [3] who showed that centrifuging at 3000 rpm was an improvement on Ridley and Hawgood's technique which centrifuged at 2000 rpm for 1 minute [2]. The centrifugal time at any given speed is also critical, since ova and cysts may remain in suspension if the sample is not centrifuged for long enough. However, there is a balance, as although the recovery of parasites was best if the samples were centrifuged at 3,500 rpm for 10 minutes, an increased amount of debris was observed, making the deposit more difficult to examine. It was also noted that there was no degradation of the ova and cysts when centrifuging at 3000 rpm for 10 minutes. This study showed that centrifuging at 3000 rpm for 3 minutes improved parasite recovery compared to the Ridley Allen Method which centrifuged at 3000 rpm for 1 minute.

When considering the health and safety aspects and ease of use of the available concentration systems, the standard Ridley-Allen sedimentation technique is open, is a multi-stage process and uses several pieces of apparatus (centrifuge tubes, boiling tubes, brass sieve and a collection receptacle) all of which require cleaning after use. Commercial kits have the advantage of being disposable enclosed systems which minimize the number of pieces of apparatus used and reduce the need for cleaning. However, like the Ridley Allen method, they require formalin which is an irritant and ethyl acetate which is flammable. Commercial kits are enclosed systems and have an air/liquid seal to prevent the release of hazardous material plus a lock to ensure that the mixing chamber and inbuilt filter are removed together for safe disposal after centrifugation. The risk of solvent exposure is further minimised by performing the procedure in a safety cabinet. However, solvents cannot be omitted as to do so results in reduced recovery of ova, cysts and larvae from faecal specimens [3].

Our review of the methods used in commercial kits for faecal concentration showed that the majority of manufacturers did not adhere to the recommended procedure of Ridley and Allen although they claim that their kit has comparable sensitivity to it. Our analysis of participants’ reported concentration procedures showed that the majority did not follow the manufacturer’s instructions.

### Conclusions

A concentration method is essential to increase the chance of finding parasite stages in a faecal specimen. This study has shown that the preservative used, solvent used, differences in centrifugation time and speed, and pore size of the sieve all affect the recovery. It also showed that the modified Ridley Allen method was optimised when using ethyl acetate with triton X instead of ether and the centrifugation time was increased from one to three minutes. Manufacturers and users of concentration kits alike should adhere to the recommendations given below to increase the recovery of parasite stages from faecal samples, not least because failing to do so will have an adverse effect on their detection in clinical specimens.

### Recommendations

Our recommendation for the concentration method is that approximately 1 gram or a pea-sized amount of faeces should be concentrated and that 10% formalin in water should be used instead of formalin in saline. A surfactant (Triton X 100) should be added when using ethyl acetate as a solvent. The most effective concentration of Triton X 100 is 0.1%. The sample must be sieved after adding it to formalin water using a pore size no greater than 0.5mm. The sample must be vortexed for at least 15 seconds after the addition of ethyl acetate and Triton X. Adequate centrifugal force must be used, i.e. 3000 rpm which equates to 1,200g. This confirms the centrifugal speed recommended in the modified Ridley Allen method [3]. A centrifugal time of 3 minutes is recommended. Three drops of saline should be added to the deposit prior to examination. Failing to do so results in the deposit being too dense to examine.
